# Effectiveness of “rescue saccades” on the accuracy of tracking multiple moving targets: An eye-tracking study on the effects of target occlusions

**DOI:** 10.1167/jov.20.12.5

**Published:** 2020-11-16

**Authors:** Shiva Kamkar, Hamid Abrishami Moghaddam, Reza Lashgari, Lauri Oksama, Jie Li, Jukka Hyönä

**Affiliations:** Machine Vision and Medical Image Processing Laboratory, Faculty of Electrical and Computer Engineering, K. N. Toosi University of Technology, Tehran, Iran; Brain Engineering Research Center, Institute for Research in Fundamental Sciences (IPM), Tehran, Iran; Institute of Medical Science and Technology, Shahid Beheshti University, Tehran, Iran; Finnish Defence Research Agency, Human Performance Division, Järvenpää, Finland; Institutes of Psychological Sciences, Hangzhou Normal University, Hangzhou, China; Department of Psychology, University of Turku, Turku, Finland

**Keywords:** multiple target tracking, occlusion, rescue saccades, eye movements

## Abstract

Occlusion is one of the main challenges in tracking multiple moving objects. In almost all real-world scenarios, a moving object or a stationary obstacle occludes targets partially or completely for a short or long time during their movement. A previous study (Zelinsky & Todor, 2010) reported that subjects make timely saccades toward the object in danger of being occluded. Observers make these so-called “rescue saccades” to prevent target swapping. In this study, we examined whether these saccades are helpful. To this aim, we used as the stimuli recorded videos from natural movement of zebrafish larvae swimming freely in a circular container. We considered two main types of occlusion: object-object occlusions that naturally exist in the videos, and object-occluder occlusions created by adding a stationary doughnut-shape occluder in some videos. Four different scenarios were studied: (1) no occlusions, (2) only object-object occlusions, (3) only object-occluder occlusion, or (4) both object-object and object-occluder occlusions. For each condition, two set sizes (two and four) were applied. Participants’ eye movements were recorded during tracking, and rescue saccades were extracted afterward. The results showed that rescue saccades are helpful in handling object-object occlusions but had no reliable effect on tracking through object-occluder occlusions. The presence of occlusions generally increased visual sampling of the scenes; nevertheless, tracking accuracy declined due to occlusion.

## Introduction

Multiple target tracking (MTT) is critical for many daily activities. For example, we track pedestrians and obstacles to steer ourselves through a crowded sidewalk. In addition, when playing or watching group sports, such as basketball, football, or volleyball, we need to track the players’ whereabouts when they move on the court. According to the literature, even 2-year-old infants are able to track more than one moving object (Cheng et al., 2019), and this ability improves as they grow older. In cognitive science, MTT experiments are used to study behavioral and/or neural aspects related to dynamic attention, short-term memory, and eye movements (for reviews, see Hyönä et al., 2019; Kamkar et al., 2020; Meyerhoff et al., 2017).

One of the most important challenging situations in MTT is target occlusion. Targets in almost all real-world scenarios are involved in temporary occlusions. An occluder (a physical object such as a wall) or another moving object can occlude them either partially or completely for a short or long time. In many MTT studies, researchers manipulate object movement to avoid occlusions (e.g., Crowe et al., 2019; Lapierre et al., 2017; Liu et al., 2005; Vater et al., 2016; Vater et al., 2017; Wahn et al., 2016; Wang et al., 2016; Wu & Wolfe, 2018). They make their targets bounce off when they are about to collide with each other. However, some studies have shown that tracking is robust to occlusion in both classic multiple object tracking experiments using identical targets (Horowitz et al., 2006; Viswanathan & Mingolla, 1998) and in those that use stimuli more similar to ones appearing in real world scenes (Lochner & Trick, 2014; Zelinsky & Neider, 2008). These studies show that observers actively allocate their attentional resources to track objects that are under occlusion. Functional magnetic resonance imagery (fMRI) studies have shown that similar cortical regions are involved in processing a moving object regardless of the object being visible or occluded (Olson et al., 2004). Despite active tracking of occluded targets, performance deteriorates a little due to target occlusion (Flombaum et al., 2008).

Despite the ubiquitous nature of object-object occlusions in real-world scenarios, only few studies have considered effects of this type of occlusions on target tracking (Viswanathan & Mingolla, 1998; Zelinsky & Neider, 2008; Zelinsky & Todor, 2010). Instead, a more common practice to study occlusion in MTT has been to use an obstacle, usually a rectangle that covers a part of the scene and occludes objects that move behind it (Flombaum et al., 2008; Keane & Pylyshyn, 2006; Olson et al., 2004; Scholl & Pylyshyn, 1999). It has been shown that tracking through darkness (a black occluder) improves performance during single object tracking for both infants and adults (Hespos et al., 2009). It is not clear if that is true in MTT also. Some researchers studied how the way objects are going to disappear and reappear again can affect MTT performance (Scholl & Pylyshyn, 1999). For example, gradual disappearance of an object while moving behind the occluder or gradual appearance when it comes out are shown to be useful for tracking through occlusion. Tracking accuracy is higher in this condition compared with the situation in which target(s) disappear/reappear in other ways, for example, instantaneously (Scholl & Pylyshyn, 1999). However, there is some evidence in support of the view that occlusion cues are detrimental for tracking (Horowitz et al., 2006). Moreover, Horowitz et al. (2006) found that simultaneous disappearance of multiple targets leads to better performance compared with asynchronous disappearance.

Occlusion has been a suitable paradigm to study whether observers use motion prediction in tracking. Researchers in this area have manipulated the location of disappearing or reappearing targets after occlusion (Keane & Pylyshyn, 2006). Franconeri et al. (2012) argue against using motion extrapolation and postulate that a proximity heuristic helps observers to handle the occlusion challenge. Participants’ performance is always better when the target reappears near the location where it has disappeared before occlusion.

Zelinsky and Todor (2010) studied participants’ eye movements while doing a pseudo-natural MTT task: following multiple sharks in a simulated underwater scene. They found that subjects make timely saccades toward a target that is in danger of being lost (because of occlusion or crowding). They called these saccades “rescue saccades,” (RS) as they are assumed to be used to prevent target swapping. This raises the question whether these saccades are successful and indeed improve tracking accuracy. Vater et al. (2017) also demonstrated the existence of such saccades while studying gaze behavior during target collisions against the bordering frame as well as in crowding of targets and distractors. However, they argue that these saccades might be harmful because they disrupt the continuous flow of visual information. Thus, it seems clear that observers make saccades toward targets that are about to be occluded or in danger of being occluded. However, it is less clear whether these eye movements serve a helpful function in tracking or whether they are in fact harmful. In the present study, our main aim was to examine whether eye movements programmed toward occluded targets are functional in MTT.

In our study, we investigated effects of occlusion on MTT with a particular emphasis on the question whether so called RS are actually helpful or not. We recorded observers’ eye movements while they tracked video-recorded natural movements of zebrafish larvae available online (Wang et al., 2017). In the videos, the larvae move about freely in a circular container. Object-object occlusions are an inseparable part of natural motion and exist in our stimuli. We also added a doughnut-shaped occluder to some videos in order to create an object-occluder condition. We considered four different conditions. First, only object-object occlusions (OB) occur for the targets. Second, only object-occluder (OC) occlusions occur for the targets. Third, both object-object and object-occluder (OBC) occlusions occur for the targets. It should be noted that OBC is the most similar condition to the real-world scenarios because targets experience both types of occlusions (OB and OC). Fourth, no occlusions (NO) occur for the targets. We also manipulated target set size with an easy (two targets) and a more difficult (four targets) condition. Therefore, we used a four (occlusion type) by two (set size) within-participants design. We analyzed participants’ eye behavior with a particular emphasis on the effect of RSs on tracking accuracy.

To compare OB against OC occlusion, we considered two criteria for target selection with the idea of excluding factors that could potentially influence tracking accuracy. First, the number of occlusions for the targets is matched between the OB and OC conditions. Second, the average length of target trajectories is equated for all conditions.

The following predictions were made. If occlusions are detrimental to MTT, the NO condition should produce better performance than all the occlusion conditions. If fixations made on to-be-occluded targets are beneficial for tracking, occluded targets receiving a fixation in the temporal vicinity of occlusion should be tracked better than occluded targets not receiving a fixation. In general, targets that receive RSs are expected to be tracked more accurately than those without RSs. With respect to performance differences among the three occlusion conditions, the condition where targets are occluded both by other moving objects (targets or distractors) and by the occluder is predicted to produce the poorest tracking performance. The OB occlusion is predicted to yield a better performance than the OC occlusion, because, in the former case, the target is never completely occluded, which is the case in the OC condition. If saccades to occluded objects are used to maintain target tracking, the most difficult occlusion condition should produce the greatest number of such saccades and the easiest occlusion condition the least number of such saccades. Finally, the aforementioned predictions should hold particularly with the larger set size (four targets). The study was preregistered in Open Science Framework (https://osf.io/xk9mn).

## Method

### Participants

Thirty students (22 women and 8 men) from the University of Turku participated in the experiment. Mean and SD of their age were 27.67 and 7.35, respectively. All participants had normal or corrected-to-normal vision and were naïve with respect to the purpose of the experiment.

### Apparatus

The stimuli were presented on a 24-inch LED monitor with 1920 × 1080 resolution and 99 Hz refresh rate. Participants used a head-chin rest during the experiment. The distance between the eye and the monitor was set to 67 cm. Subjects’ eye movements were recorded by an Eyelink 1000 eye-tracker (SR Research, Ottawa, Ontario, Canada) using a 35 mm lens and a 1000 Hz sampling rate (with 3 subjects the sampling rate was set to 500 Hz, which is taken into account in the analyses). The distance between the camera and the eyes was set to 57 cm.

### Stimuli

We used recorded videos of natural movement of multiple zebrafish larvae that moved about within a circular container. The videos are available online (moving zebrafish larvae segmentation and tracking dataset^1^; Wang et al., 2017). From among the different videos available in the dataset, we selected those that were similar with respect to the scene and the scale of the fishes (videos # 1, 7, and 10). For the manipulation of set size (two or four targets), we needed eight objects in the scene so that at least four of the objects are distracters. However, the selected videos contained only four larvae. To get unique movies with eight larvae, we first cut the videos to shorter parts (each about 7.8 seconds), then rotated them using different rotation angles and finally merged them two by two. The background area (the circular container) was set to gray. The diameter of the circular container was 14.46 degrees of visual angle and the size of the minimum bounding rectangle of each straight larva was 2.57 by 0.43 degrees of visual angle.

A doughnut-shape occluder was added to the center of the videos in the OC and OBC conditions. The occluder's texture was made by mixing all the larvae's texture. The thickness and the diameter of the minimal bounding circle of the occluder were 1.70 and 11.05 degrees of visual angle, respectively.

**Figure 1.**
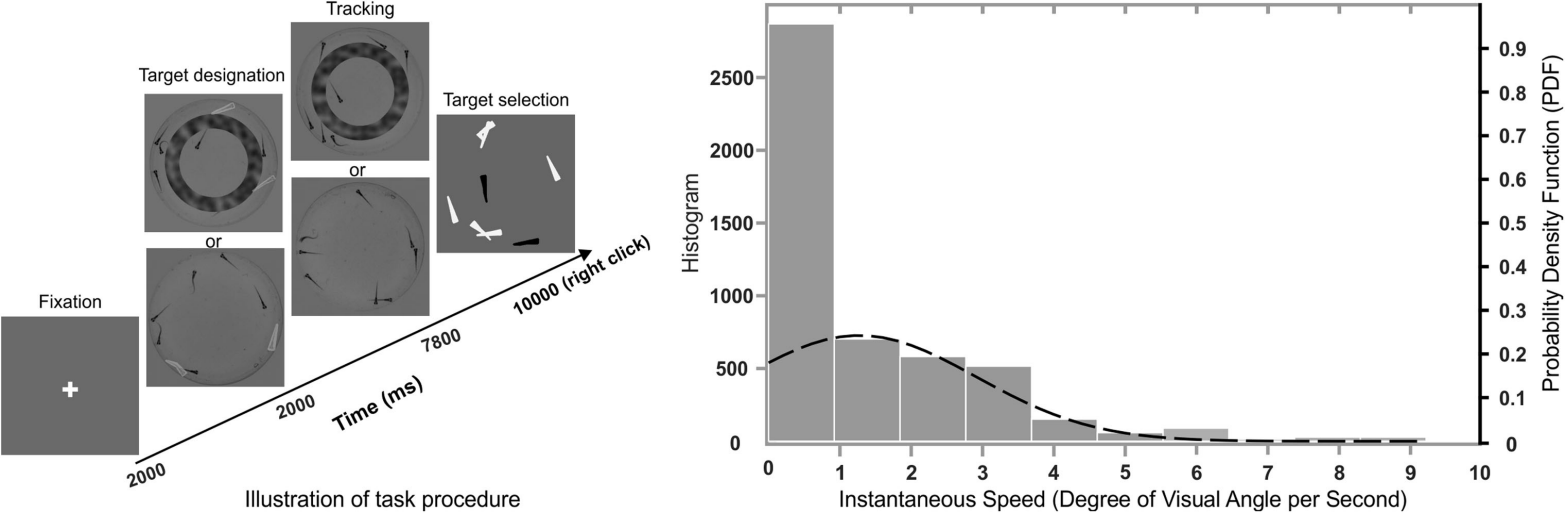
Left: Timeline of the experiment. In the first stage, a fixation cross was presented in the middle of the screen for 2 seconds. During the target designation phase, two or four larvae turned red (gray in this illustration) either with or without the doughnut-shape occluder and were flashed for 2 seconds. The participant was to track the flashed targets for 7.8 seconds either with or without the doughnut-shape occluder, after which all objects were masked. Finally, during the target selection phase all objects stopped moving and turned to white; the participant had to click with the computer mouse on the targets, which then turned to black. Right: The distribution of instantaneous speed of larvae's natural movement.

The motion trajectory and the number of occlusions varied for different larvae in different videos because of their natural movement. The larvae's natural movements are unpredictable and bursty. Their speed in each second follow a truncated normal distribution with mean mu = 1.3 and SD sigma = 1.6 degrees of visual angle per second (Figure 1, right panel). Their motion is also uncorrelated. To prove that, we assessed the degree of similarity in target motion by using the method proposed by Velloso et al. (2017). An average correlation in target motion during each trial was computed for all target pairs. A corresponding number of random target trajectories was then created that were identical in length with the target trajectories. A trial-wise average correlation was computed for each pair of random trajectories. This resulted in 160 correlations for target trajectories and 160 correlations for random paths. The difference in correlations was then tested with a 1-way ANOVA, which showed that the target trajectories did not significantly differ from random trajectories (*p* = 0.20). The mean correlation of target trajectories was 0.52 (SD = 0.20) and that of random trajectories 0.44 (SD = 0.25). After the experiment, participants also expressed that the target movement felt unpredictable, which made them rather hard to track.

An example video of each of the four conditions is provided in Supplementary Videos 1, 2, 3, and 4 (the targets in the example videos are shown in red only for illustrative purposes. In the actual experiment they were not red). The average number of occlusions that a target was involved in is similar for the occlusion conditions of OB and OC and across the two set sizes ($F(3,\;57)\;=\;0{.41,\;\eta }_{p\;\;}^{2}=\;0.004,\;p\;=\;0.75$). Further, the average length of target trajectories (in pixels) for the different videos was not significantly different between the NO, OB, OC, and OBC conditions and across the set sizes ($F(7,\;133)\;=\;1{.01,\;\eta }_{p}^{2\;\;}=\;0.05,\;p\;=\;0.43;$ Table 1).

**Table 1.** Mean and standard error of mean (*SEM*) of targets’ number of occlusions and trajectory length in all conditions.

	Average number		Average length of	
	of target		target's trajectory	
	occlusions		in pixels	
Condition	Mean	*SEM*	Mean	*SEM*
NO2	–	–	435	48
NO4	–	–	308	34
OB2	1.65	.09	391	41
OB4	1.70	.11	406	38
OC2	1.58	.10	365	45
OC4	1.56	.10	389	35
OBC2	4.53	.21	445	38
OBC4	3.75	.28	453	69

### Procedure and design

We used a four (occlusion types = NO, OB, OC, and OBC) by two (target set size = 2 or 4) within-participants design. Thus, there were 8 conditions each with 20 trials. The 160 trials were presented in 4 blocks each with 40 trials. The trials in each block were selected randomly from different conditions and were shuffled before presenting them to the subjects. Each block started with a nine-point calibration of the eye-tracker followed by a validation and drift check. Each trial started with a 2-second fixation on a cross in the middle of the screen. Then, 2 or 4 larvae turned red and were flashed for 5 times during 2 seconds (the target designation phase). Then, 8 larvae started moving naturally for 7.8 seconds (the MTT phase). Finally, all objects stopped moving and were masked by a white polygon that bounds them (see Figure 1). Then the participants had to click on the targets using the computer mouse (see Figure 1 for the timeline). The clicked targets turned to black. The selected items and their corresponding response times were registered. The experiment was carried out in a dim-lit room to help participants to focus on the task. Eye movements of the right eye were recorded for the entire duration of each block. There was a short rest after each block; the whole experiment lasted for about 1 hour for each participant.

## Results

We used four (occlusion type = NO, OB, OC, and OBC) by two (target set size = 2 or 4) repeated measures ANOVAs (both classical and Bayesian) to test the main effects of occlusion and set size as well as their interaction. For all tests, *p* = 0.05 is considered statistically significant. In case of a significant main effect of occlusion, we applied *t*-tests with Bonferroni correction to find out the significantly different pairs. In what follows, we first report the results for the effects of occlusion type and set size on performance accuracy and eye behavior, followed by analyses testing the usefulness of saccades executed toward occluded targets.

### Performance accuracy

**Figure 2.**
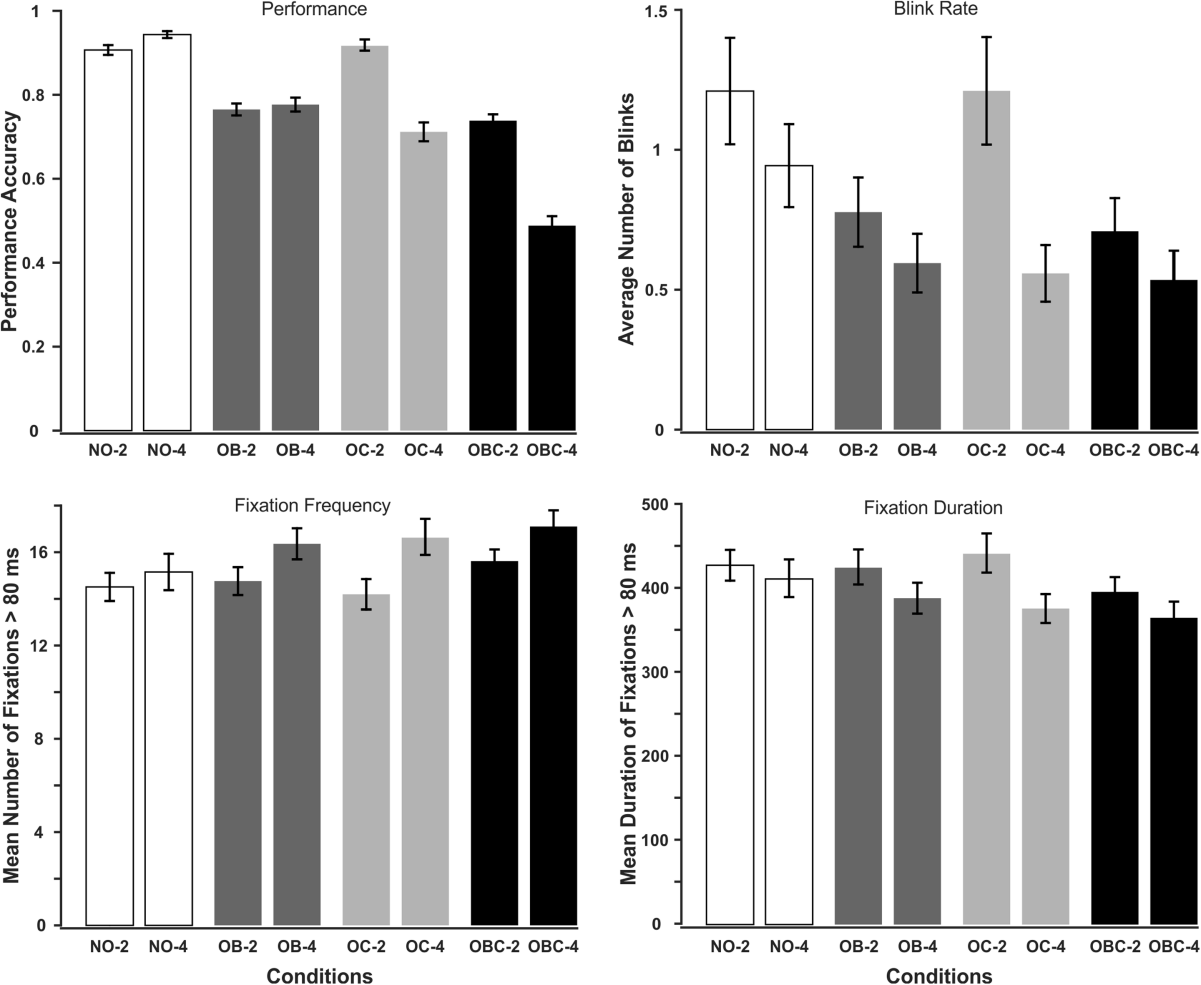
Top left: Average tracking performance, top right: blink rate, bottom left: fixation frequency, bottom right: fixation duration. Error bars show standard error of mean. NO = no occlusions; OB = object-object occlusions; OC = object-occluder occlusions; OBC = object-object + object-occlusion occlusions.

Accuracy for each trial is considered either one or zero, one if all targets were marked correctly or zero if at least one target was missed.^2^ We then calculated the average performance for the eight conditions (see Figure 2). ANOVA showed significant main effects for both occlusion type and set size as well as their interaction (Table 2). All pairwise comparisons between the occlusion types, except between OB and OC, were significantly different (t(59)  >  5.6728,  p  <  0.001). Accordingly, any type of occlusion deteriorates the tracking performance compared with the NO condition. The OBC condition, where both types of occlusion occurred, was the hardest condition with poorest performance. As expected, the accuracy in four-target cases was lower than their corresponding two-target cases for the OC and OBC conditions. On the other hand, for the NO condition, performance was near ceiling, and in the OB condition, set size did not affect the accuracy. These differences between the occlusion conditions for the set size effect explains the significant interaction.

**Table 2.** The ANOVA results for accuracy, fixation frequency, fixation duration, blink rate, number of target visits, number of updated targets, and number of nontarget visits.

			Fixation	Fixation	Blink	Target	Updated	Nontarget
Variables		Accuracy	frequency	duration	rate	visits	targets	visits
**Occlusion**	df	3, 87	3, 87	3, 87	3, 87	3, 87	3, 87	3, 87
	F	163.5354^***^	17.6754^***^	11.4179^***^	19.8014^***^	5.2934^**^	6.5643^***^	8.4436^***^
	$\eta_{p}^{2}$	0.8494	0.3787	0.2825	0.4058	0.1544	0.1846	0.2255
	***BF_10_***	>100	>100	>100	>100	.087	.085	4.782
**Set-Size**	df	1, 29	1, 29	1, 29	1, 29	1, 29	1, 29	1, 29
	F	99.6620^***^	25.6098^***^	27.6346^***^	26.3728^***^	60.3576^***^	79.1410^***^	101.8869^***^
	$\eta_{p}^{2}$	0.7746	0.4690	0.4879	0.4763	0.6755	0.7318	0.7784
	***BF_10_***	>100	>100	>100	>100	>100	>100	>100
**Occlusion** × **Set-Size**	df	3, 87	3, 87	3, 87	3, 87	3, 87	3, 87	3, 87
	F	53.6317^***^	11.8781^***^	6.5906^***^	10.5887^***^	16.1473^***^	41.8407^***^	33.8882^***^
	$\eta_{p}^{2}$	0.6490	0.2906	0.1852	0.2675	0.3577	0.5906	0.5389
	***BF_10_***	>100	10.6049	6.6759	77.2277	2.85	23.2143	>100

**p  <  0.01; ***p  <  0.001.

### Fixations and blinks

ANOVA showed significant main effects of occlusion and set size and their interaction for fixation frequency and duration (see Figure 2; Table 2). Fixation frequency differed in all pairwise comparisons (|t(59)|  >  2.9809,  p  <  0.005) except between OB and OC. The highest frequency was for the OBC condition and the lowest one for NO. Moreover, participants made more fixations when tracking four than two targets. The interaction primarily reflects the fact that the set size effect is negligible for the NO condition. On the other hand, average fixation duration was shorter for OBC than other occlusion conditions (t(59)  >  3.9831,  p  <  0.001), with the other conditions not differing significantly from each other. Moreover, participants made shorter fixations when tracking four than two targets. The interaction reflects the fact that the set size effect was smallest for NO and largest for OC.

Similarly, the main effect of occlusion and set size were significant for blink rate, as was their interaction. All pairwise comparisons for the occlusion conditions were significant except between OB and OBC (|t(59)|  >  3.1475,  p  <  0.005). Moreover, participants blinked less when tracking four than two targets. The interaction reflects the fact that the set size effect was particularly robust for the OC condition.

In sum, participants blinked the most and made the least fixations that lasted for the longest time during the NO condition. On the other hand, when targets were occluded with other objects and an occluder (OBC), they blinked less and increased the number of fixations that lasted for shorter duration.

### Target visits

**Figure 3.**
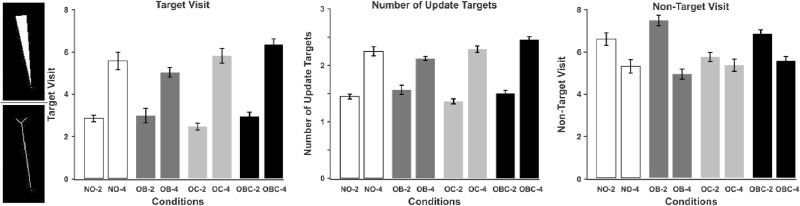
The left-most part of the figure shows a sample larva's skeleton (bottom) made from its minimal bounding polygon (top). The left, middle, and right panels depict average number of target visits, updated targets and nontarget visits, respectively. OB = object-object occlusion; OC = object-occluder occlusion; OBC = object-object + object-occluder occlusion. Error bars show standard error of mean.

We also calculated the number of target and nontarget visits as well as number of updated targets. For target/nontarget visits, we computed the number of times the targets/nontargets were overtly attended by fixating them; for the updated targets, we considered the number of unique targets that were overtly attended at least once (see also Oksama & Hyönä, 2016). A target/nontarget was considered to be visited when the distance between gaze position and the target's/nontarget's skeleton was less than three degrees of visual angle. This threshold was determined by regarding the eye-tracker accuracy, the distance of a typical larva's border from its skeleton and the area that projects to the fovea. Target's skeleton was calculated after applying some morphological operations on larva's minimal bounding polygon (see the left-most part of Figure 3). Information related to the minimal bounding polygon of all larvae in all frames were extracted using the Label Me annotation tool.^3^

A similar pattern of results emerged for both target visit measures. The main effect of occlusion type, the main effect of set size, and their interaction were all significant (see Table 2). Increase in set size resulted in more target visits and updated targets, because there were more targets to visit in set size four than set size two. The main effect of occlusion type is due to OBC producing significantly more target visits (|t(59)|  >  4.0367,  p  <  0.001) and updated targets (|t(59)|  >  3.7561,  p  <  0.001) than the other conditions. The interaction was due to the set size effect being largest for OBC and smallest for OB. In addition, in nontarget visits, we obtained a significant main effect of occlusion type and set size as well as their interaction. Nontargets in the OC condition are visited significantly less than in OB and OBC (|t(59)|  >  4.269,  p  <  0.001) which explains the main effect of occlusion. The set size effect is the largest for OB and smallest for OC, which is the reason behind the significant interaction of the factors.

### Pupil dilation

**Figure 4.**
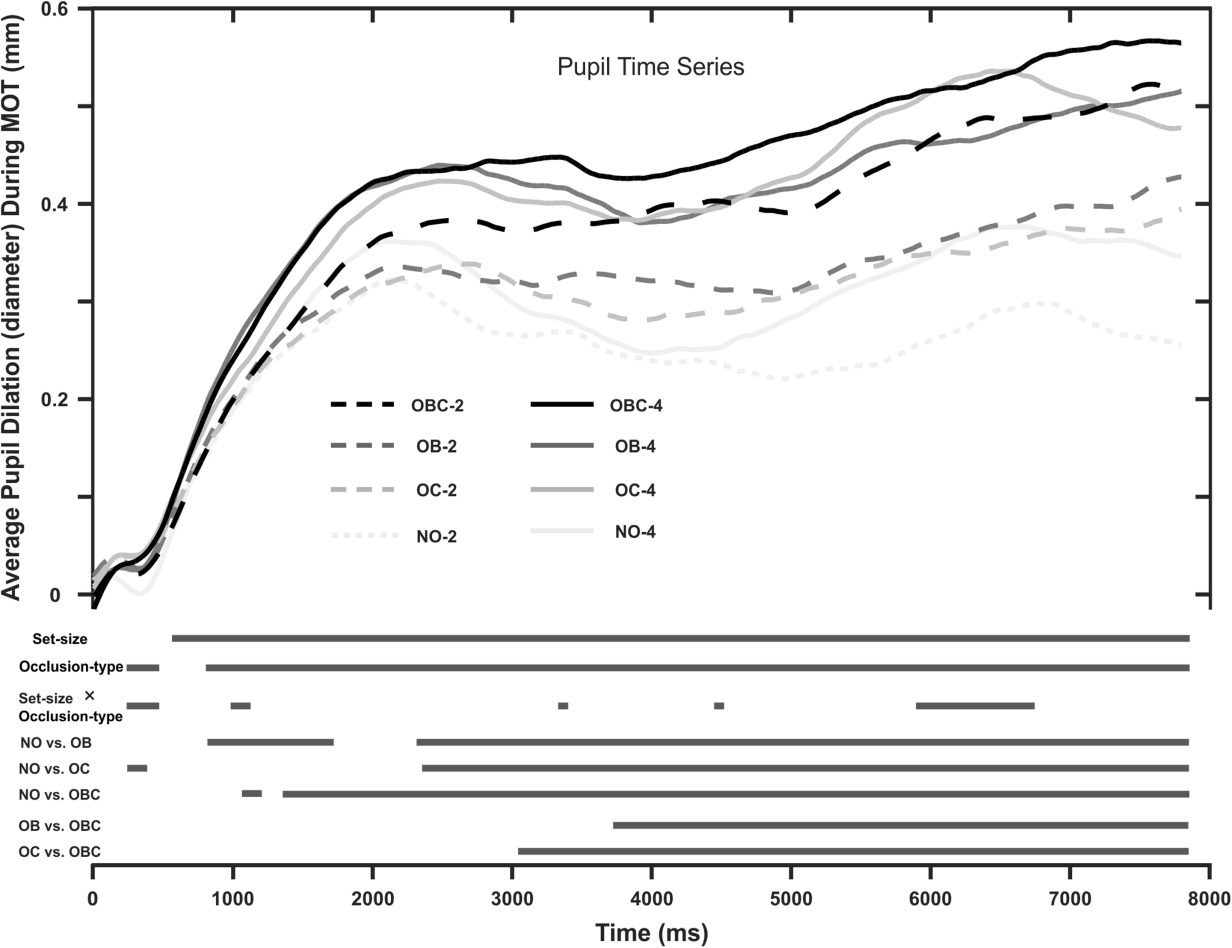
Average pupil size difference to the baseline during tracking (the upper curves). The bars below the curves show the time points during which the main effect of occlusion type and set size as well as their interaction were statistically significant. It also shows the time points during which the pairwise comparisons between the occlusion conditions were significant. OB = object-object occlusion; OC = object-occluder occlusion; OBC = object-object + object-occluder occlusion.

We also analyzed pupil dilation as an index of cognitive effort invested during tracking. In order to do so, we first preprocessed raw pupil data using the algorithm proposed by Kret and Sjak-Shie (2019). For each trial, we considered mean pupil diameter in the last 100 ms of the target designation phase as the baseline for the following MTT phase. Figure 4 depicts how pupil diameter on average varied in different conditions during tracking. The main effect of set size and occlusion type were significant for almost the entire MTT phase (see the dark lines toward the bottom of Figure 4). Among the different occlusion conditions, pupil size in OB and OC were similar. Other comparisons were significantly different in pupil diameter according to the *t*-tests with Bonferroni correction. This suggests that NO is the least cognitively demanding condition and OBC is the most demanding one. OB and OC are between these two. This suggests that cognitive demand is similar whether targets are followed through OB occlusion or OC occlusion. Pupil size was also bigger for trials with four rather than two targets. Finally, during a time period between 6 and 7 seconds the occlusion type × set size interaction was significant. This appears to be due to the pupil size temporarily increasing for the OC condition with four targets.

### Are rescue saccades helpful in tracking?

**Figure 5.**
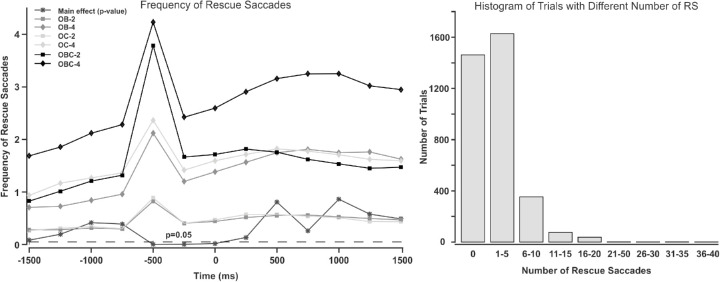
The right panel shows the histogram of the number of trials with RSs. The left panel depicts the *p* value of the main effect of RS (gray line with stars) in target accuracy in different time windows (a 1-second window was used, 500 ms to either side from the center of window). Lines with squares and diamonds show the frequency of RSs for the different occlusion conditions in each time window. OB = object-object occlusion; OC = object-occluder occlusion; OBC = object-object + object-occluder occlusion. Error bars show standard error of mean.

In order to determine the usefulness of saccades executed toward occluded targets, we analyzed whether tracking accuracy was better with than without such RSs. We considered two criteria to account a saccade as an RS. First, it should land near to a to-be-occluded target. The distance between the saccade's landing location and the target's skeleton should be a maximum of three degrees of visual angle. Second, the saccade should happen within a specified time window relative to the time when a target starts to be occluded (occlusion onset). In the Zelinsky and Todor study (2010), saccades within 800 ms preceding the end of occlusion were accounted as RSs. However, Vater et al. (2017) considered saccades within an interval of 200 ms before collision. We also considered the start time of occlusion rather than the end time, as in natural videos, the targets spent variable times behind the occluder. We defined a 1-second time window (500 ms to either side from the center of window). We slid this window in 250 ms steps from 2000 ms preceding to 2000 ms following occlusion onset. Figure 5 (left panel) shows how *p* values of the main effect of RSs on target accuracy changes in different time windows (the line with star markers). As is evident from the figure, RSs executed within the 500 ms time window before occlusion onset significantly improve target tracking accuracy. More details on this analysis are provided in the section Effect of rescue saccades on target accuracy.

The lines in Figure 5 (left panel) show the frequency of RSs within each time window for the different occlusion conditions. A three (OB, OC, and OBC) by two (set size 2 and 4) ANOVA shows a significant main effect of set size ($F(1,\;29)=120.6629,\;\eta_{p}^{2}=0.8062,\;p<0.001,BF_{10}>100$) and occlusion type ($F(2,\;58)=175.2643,\;\eta_{p}^{2\;}=2.8819,\;p<0.001,BF_{10}>100$) in the frequency of RSs. The interaction of the two variables was not significant ($F(2,\;58)=3.0709,\;\eta_{p}^{2}=0.0957,\;p=0.054,BF_{10}=0.7668$). The main effect of occlusion is due to OBC producing more RSs than the other two occlusion conditions (OB and OC) that did not differ from each other. This makes sense, as OBC contained more occlusion events than the other two conditions, for which the number of occlusions was equated (see Table 1). Moreover, participants made more RSs when tracking four rather than two targets. It is noteworthy that this effect does not reflect the number of occlusions, as it was matched across the two set sizes for OB and OC (see Table 1). Rather, it hints at the possibility that RSs are especially needed with large set sizes. Obtaining a set size effect despite a similar number of occlusions in the OB-2, OB-4, OC-2, and OC-4 conditions is due to an increase in the frequency of target visit by increasing set size. This is in line with previous research, which showed that observers frequently switch fixations between each target and the centroid of multiple targets during multiple object tracking (MOT), so that there are more saccades to targets as the set size increases (Hyönä et al., 2019).

In order to see whether RSs are helpful, we considered their general effect as well as a local effect. Making RSs can have a general effect on the trial's accuracy and/or a local effect on the to-be-occluded target's accuracy. To study the effect of RS on the trials’ accuracy, we divided all trials in each condition to two or more groups, according to the number of RSs that happened in them. Then we compared the trials’ accuracy in the groups to examine first, if the number of RSs can significantly affect tracking accuracy, and second, whether making an RS, in general, can improve tracking accuracy compared with when no RSs are made during the trial.

Moreover, to investigate the effect of RSs on the accuracy of the to-be-occluded targets, we divided targets in each trial into two groups: (1) target(s) that have received an RS in that trial and (2) target(s) that have not received an RS toward them. We compared these two groups across all trials of all subjects in each condition separately. Results are given in the section Effect of rescue saccades on target accuracy.

#### Effect of rescue saccades on trial accuracy

We computed the number of RSs in each trial and plotted their histogram (see the right panel of Figure 5). Most of the trials had at least one RS.

**Figure 6.**
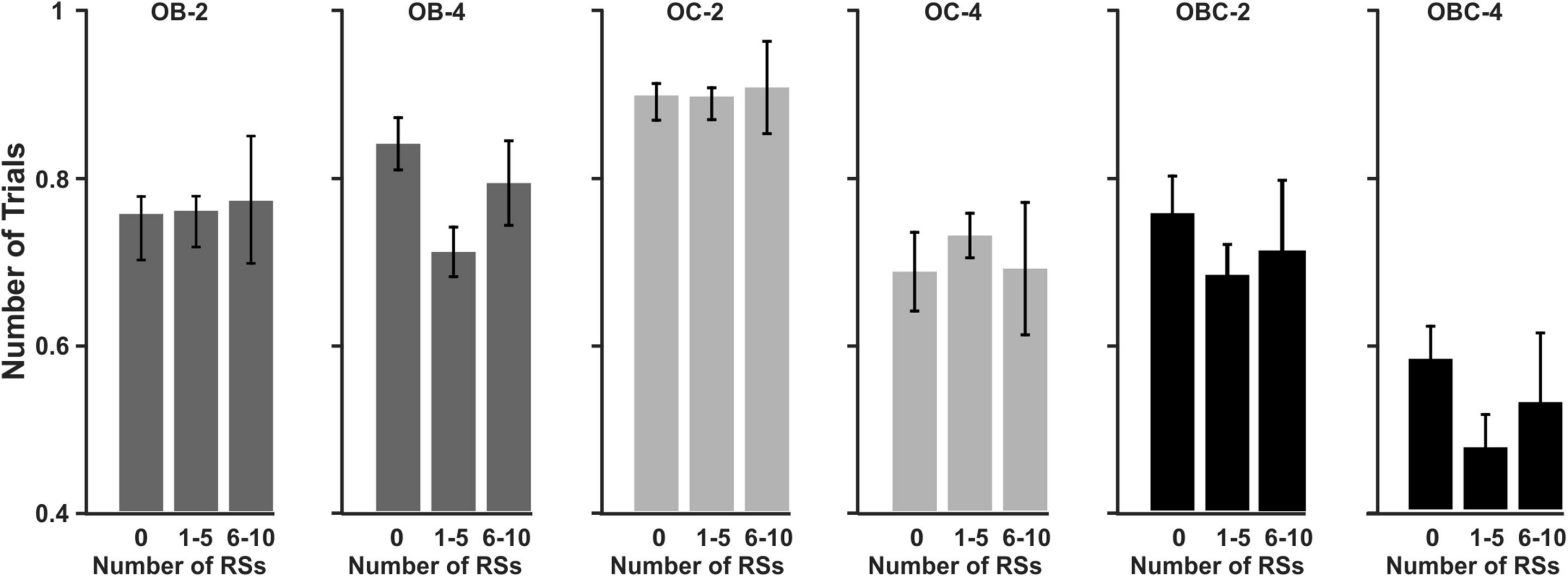
Tracking accuracy for trials with different number of RSs as a function of occlusion type and set size (2 vs. 4). OB = object-object occlusion; OC = object-occluder occlusion; OBC = object-object + object-occluder occlusion.

In order to see whether RSs are helpful, we conducted two sets of analyses. In the first analysis, we divided the trials into three categories (no RSs, 1–5 RSs, and 6–10 RSs) and compared tracking accuracy between them. We excluded trials with > 10 RSs, because their number was very low. Figure 6 depicts the tracking accuracy for the three categories separately for the different occlusion types. A three (OB, OC, and OBC) × 3 (0, 1–5, and 6–10 RSs) × 2 (set size 2 and 4) ANOVA showed significant main effects of occlusion $\left(F\left(2,\;58\right)=11.373,\;\eta_{p}^{2\;}=0.4155,\;p<0.001,BF_{10}>100\right)$ and set size ($F(1,\;29)=15.183,\;\eta_{p}^{2}=0.4870,\;p<0.005,BF_{10}>100$). The occlusion × set size interaction was significant too ($F(2,\;58)=10.478,\;\eta_{p}^{2}=0.3958,p<0.001,BF_{10}>100$). Here, we focus on the effects involving RS as a factor. None of these effects proved significant. The main effect of RS frequency was nonsignificant ($F(2,\;58)=0.976,\;\eta_{p}^{2}=0.0577,\;p=0.3876,BF_{10}=0.064$). In other words, trials with several RSs were tracked no better than trials with no RSs. Moreover, the occlusion type × RS frequency interaction ($F(4,\;116)=0.509,\;\eta_{p}^{2}=0.0307,\;p=0.7290,BF_{10}=0.0431$), the set size × RS frequency interaction ($F(2,\;58)=0.022,\;\eta_{p}^{2}=0.0014,\;p=0.9784,BF_{10}=0.0658$) and the three-way interaction ($F(4,\;116)=0.437,\;\eta_{p}^{2}=0.0267,\;p=0.7813,BF_{10}=0.0842$) were all nonsignificant.

**Figure 7.**
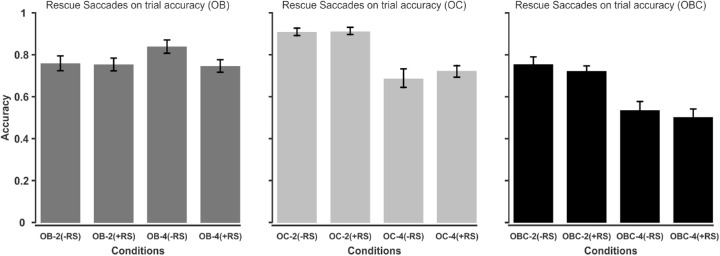
The left, middle, and right panels show the overall tracking accuracy for trials with or without RS in the OB, OC, and OBC conditions, respectively. -RS = no rescue saccades; +RS = rescue saccades; OB = object-object occlusion; OC = object-occluder occlusion; OBC = object-object + object-occluder occlusion.

In the second analysis, we divided the trials into two groups, trials with no RSs at all and trials with at least one RS. This way we could also include trials with > 10 RSs (they were excluded from the previous analysis). Figure 7 shows the results for each occlusion type separately. A three (OB, OC, and OBC) × two (set size 2 and 4) × two2 (with/without RSs) ANOVA revealed no reliable effect of RS on tracking accuracy ($F(1,\;29)=2.467,\;\eta_{p}^{2}=0.0793,\;p\;=\;0.1271,\;BF_{10}=0.2$). However, the main effects of occlusion ($F(2,\;58)\;=\;54{.415,\;\eta }_{p}^{2}=0.6522,\;p\;<\;0.001,\;BF_{10}>100$) and set size ($F(1,\;29)\;=\;47{.127,\;\eta }_{p}^{2}=0.6189,\;p\;<\;0.00$1, *BF*_10_ > 100) were significant as well as the occlusion × set size interaction ($F(2,\;58)\;=\;35{.637,\;\eta }_{p}^{2}=0.5513\;,p\;<\;\;0.001,\;BF_{10}>100$). The occlusion × RS interaction and the set size × RS interaction were not significant as well as the interaction of all three variables. In sum, both analyses showed no significant effects of RS on trials’ accuracy.

#### Effect of rescue saccades on target accuracy

**Figure 8.**
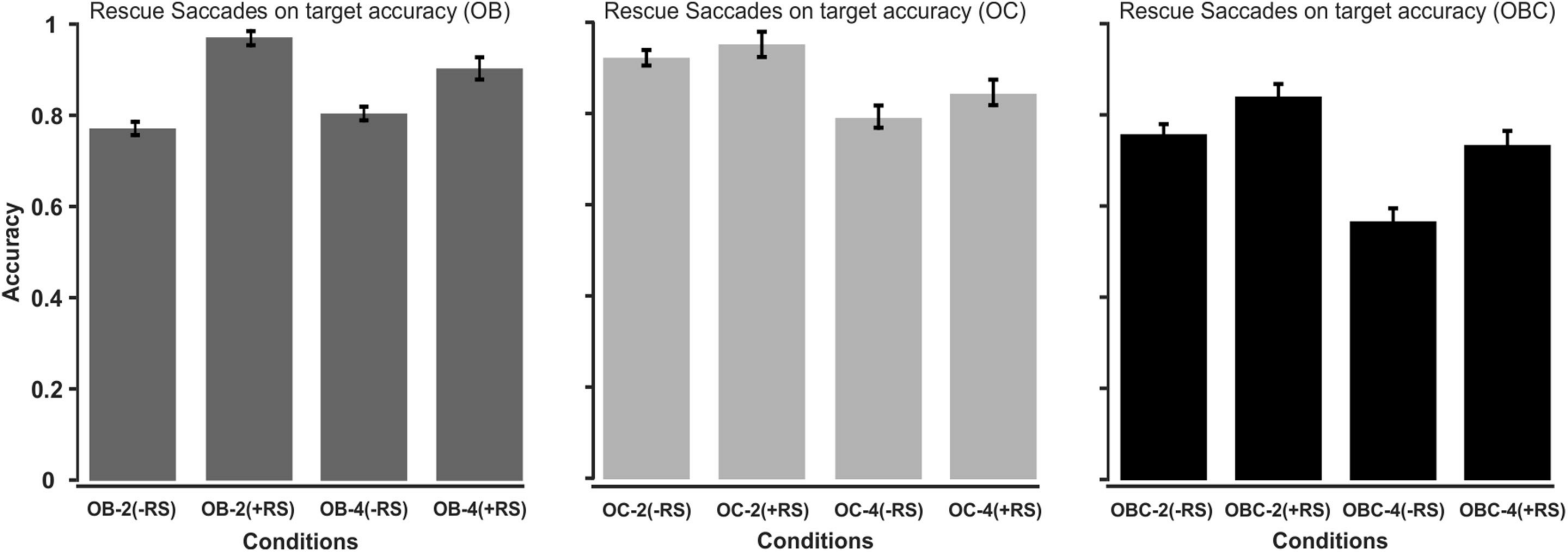
The left, middle, and right panels show the overall tracking accuracy for targets with or without RS in the 500 ms time window preceding occlusion onset for the OB, OC, and OBC conditions, respectively. -RS = no rescue saccades; +RS = rescue saccades; OB = object-object occlusion; OC = object-occluder occlusion; OBC = object-object + object-occluder occlusion.

To study the effect of RSs on target tracking accuracy, we divided the targets in all trials into two groups, targets with no RSs at all and targets with at least one RS. We compared the accuracy of these two groups of targets across all trials within the 500 ms time window before occlusion onset. As shown in Figure 5 (left panel), this was the time window where the main effect of the occurrence of RSs was significant. Figure 8 shows the results for each occlusion type separately. A three (OB, OC, and OBC) × two (set size 2 and 4) × two (with/without RSs) ANOVA revealed significant main effects of RS ($F(1,\;29)\;=\;56{.583,\;\eta }_{p}^{2}=0.6613,p<0.001,BF_{10}>100$), occlusion type ($F(2,\;58)=79.716,\;\eta_{p}^{2}=0.7334,p<0.001,BF_{10}>100$) and set size ($F(1,\;29)=113.173,\;\eta_{p}^{2}=0.7959,\;p<0.00$1, *BF*_10_ > 100). However, the main effects were qualified by the occlusion type × RS interaction ($F(2,\;58)=8.491,\;\eta_{p}^{2}=0.2266,\;p<0.00$1, *BF*_10_ = 98.1180) as well as the occlusion type × set size interaction ($F(2,\;58)=17.186,\;\eta_{p}^{2}=0.3724,p<0.001,BF_{10}>100$). The former interaction reflects the tendency for RSs improving target accuracy in OB (t(59)  =   -8.9184,  p  <  0.001) as well as OBC (t(59)  =  -6.0814,  p  <  0.001), whereas RSs had no reliable effect in OC (t(59)  =   -1.9076,  p  =  0.0613). The two-way interactions were further qualified by a significant three-way interaction ($F(1,\;29)\;=\;8{.381,\;\eta }_{p}^{2}=0.2243,\;p\;<\;0.00$1, *BF*_10_ = 23.3160). To examine the nature of the three-way interaction on more detail, we computed ANOVAs separately for the three occlusion conditions.

A two (set size 2 and 4) × two (with/without RSs) ANOVA for OB showed a nonsignificant main effect of set size ($F(1,\;29)\;=\;2{.1922,\;\eta }_{p}^{2}=0.07,\;p\;=\;0.1495,\;BF_{10}=0.273$), and a significant main effect of RS ($F(1,\;29)\;=\;87{.7557,\;\eta }_{p}^{2}=0.7516,\;p\;<\;0.001,\;BF_{10}>100$). The interaction was also significant ($F(1,\;29)\;=\;11{.0477,\;\eta }_{p}^{2}=0.2760,\;p\;<\;0.005,\;BF_{10}=48.8543$). The nature of the interaction was such that in OB the facilitatory effect of RSs was more pronounced for set size two than four (see the left panel of Figure 8).

A two (set size 2 and 4) × two2 (with/without RSs) ANOVA for OC showed a significant main effect of set size ($F(1,\;29)=46.5956,\;\eta_{p}^{2}=0.6163,p=<0.001,BF_{10}>100$), but a nonsignificant main effect of RS ($F(1,\;29)=2.9321,\;\eta_{p}^{2}=0.0918,p=0.0975,BF_{10}=0.904$). The interaction was nonsignificant ($F(1,\;29)=0.3709,\;\eta_{p}^{2}=0.0127,\;p=0.5472,BF_{10}=0.3364$). Thus, we cannot conclude that RSs improved performance in the OC condition (see the middle panel of Figure 8).

A two (set size 2 and 4) × two (with/without RSs) ANOVA for OBC showed a significant main effect of set size ($F(1,\;29)\;=\;63{.6813,\;\eta }_{p}^{2}=0.6871,p=<0.001,BF_{10}>100$), and a significant main effect of RS ($F(1,\;29)\;=\;31{.2266,\;\eta }_{p}^{2}=0.5185,\;p\;<\;0.001,\;BF_{10}>100$). In addition, the interaction was significant ($F(1,\;29)\;=\;6{.0257,\;\eta }_{p}^{2}=0.1720,\;p\;<\;0.05,\;BF_{10}=1.9789$). The interaction reflects the fact that in OBC the facilitatory effect of RSs was more pronounced for set size four than two (see the right panel of Figure 8).

To sum up the nature of the three-way interaction, in OB, the facilitatory effect of RSs was more pronounced with set size of two rather than four, whereas the opposite was true for OBC. Finally, in OC, RSs did not improve target tracking.

## Discussion

When tracking multiple moving objects in real-life visual environments (e.g. busy intersections), moving objects are constantly occluded by other moving or stationary objects. In order to perform adequately in such situations, people are required to also keep track of occluded objects. Visually attending to regions where a critical object is about to be occluded by another object (e.g. a car approaching an intersection from behind a bus) may be required to keep track of occluded objects. In order to overtly attend to a specific region in the visual environment, the observer has to make an eye movement toward that region to bring it into the foveal vision for detailed scrutiny. A prior study indeed demonstrated that observers make timely saccades toward to-be-occluded objects (Zelinsky & Todor, 2010). These saccades help obtain high-resolution visual information about the occlusion event and update position information for task-relevant objects. However, even though they are frequently made, it is not clear if they are helpful in improving tracking accuracy (Hyönä et al., 2019; Vater et al., 2017). Addressing this question was the main purpose of the present study.

We considered in the study two possible types of occlusions: target occlusion by another moving object (target or distractor) and target occlusion by a stationary occluder. We investigated effects of occlusion in multiple target tracking by using authentic videos depicting biological motion (larvae moving in a circular container). Such motion characteristics resemble more closely real-life dynamic environments than those used in the majority of prior studies on multiple object tracking. In previous studies, the speed of studied motion had typically been constant; moreover, object occlusion has been avoided. In the present study, however, the larvae moved with variable speed, but they could also remain stationary. These motion characteristics approximate those observed in real-life dynamic environments, such as traffic or team sports scenes.

We conducted three analyses on the usefulness of saccades made to the to-be-occluded targets (so called RSs, see Zelinsky & Todor, 2010); two of them were trial-based and one target-based. The trial-based analyses revealed no significant overall benefits related to the occurrence of RSs. The target-based analyses were computed across a wide time interval around occlusion onset. The results demonstrated that RSs within the 500 ms period before occlusion onset significantly improved target tracking accuracy. In other words, the timing of RSs just prior to occlusion onset is critical for obtaining a facilitatory effect. RSs were found to improve performance in the OB and OBC conditions if made in the 500 ms window before occlusion. Swapping (confusing a target with a distractor) and dropping (missing a target) are two main possible errors in MTT (Drew et al., 2013). In OB occlusion, swapping is more probable. Making a RS in such situation yields high-resolution information about the location and motion direction of the target larva, which can be helpful to avoid swapping. In the visual environment examined in the present study, swapping is quite probable, as the larvae look very much alike. On the other hand, in OC occlusion swapping is less probable (no target-distractor confusion), whereas the probability of dropping is more likely. The lack of a facilitatory effect of RSs in OC occlusion may also relate to the distribution of attention during MTT. Without occlusion, attentional resources may be evenly distributed across the targets, but the occlusion draws more attentional resources to the occluded target, and with that the overt attention as well. In OC, the occluded targets are often completely invisible (particularly when they are positioned tangential to the occluder). Thus, using RSs to update with overt attention the exact target location is not possible. Moreover, allocating attention toward the occluded target in the form of RSs happens at the expense of other targets, which may also contribute to not observing a facilitatory effect of RSs in OC. Finally, the positive effect of RSs in the OBC condition is probably due to the positive effect of RSs observed in OB.

The notion of RSs was first introduced by Zelinsky and Todor (2010). They showed that when tracking multiple moving targets, observers make saccades toward targets in danger of being occluded by other moving objects or of suffering from crowding. They chose the term “rescue saccade” to refer to the observer's gaze shift to occluding objects with the intent of preventing target swapping. Yet, they did not study their effects on tracking accuracy.

The present study provides compelling evidence for the usefulness of RSs in MOT. As discussed above, they have a facilitative effect in dealing with OB occlusions, but they cannot help much in dealing with situations where a target moves behind an occluder. Vater et al. (2017) observed a negative effect for saccades that were executed toward targets to-be-colliding to the border frame. In other words, their finding is not directly applicable to dealing with occlusions. They ascribe the detrimental effects of saccades to the disruption of continuous flow of visual information due to saccadic suppression. However, this explanation cannot be applied to the present results, as we observed RSs to be ineffective in one condition, but helpful in two other conditions.

There is also an inconsistency in results for the frequency of RSs between the present study and that of Zelinsky and Todor (2010). They found that RS frequency decreased in the four-target condition compared with two or three targets. Zelinsky and Todor (2010) considered the possibility that RSs may often be executed too late to prevent target swapping from taking place. However, our results demonstrate an increase in the number of RSs from the set size two to four. An increase in RSs as a function of set size is compatible with an analogous effect in target visits (i.e. the frequency of fixations made on targets). Zelinsky and Todor explain the decrease in RSs in set size four by limited attentional resources. With four targets, an occlusion appeared in their study, on average, about every other second, with the possibility of two occlusions appearing simultaneously. Thus, due to the high demand for RSs in set size four, the observer needs to limit the frequency of RSs – hence the decline. The present results are partly in line with the above reasoning. We found the set size effect in RS frequency to be less pronounced in the OBC condition particularly within the 500 ms period before occlusion onset (see Figure 5). It should be noted that the occlusion frequency in OBC was comparable to that in the Zelinsky and Todor study, in comparison to the OB and OC conditions, where occlusions were less frequent (see Table 1).

Apart from RSs, we also examined effects of occlusion on overall tracking behavior. We observed that participants made more but shorter fixations in general in the presence than absence of occlusions. This pattern was particularly noticeable when both OB and OC occlusions are present. These effects suggest that observers deal with occlusions by increasing the rate of sampling the visual environment with their eyes, with each sample (i.e. fixation) lasting for less time. A subset of the fixations land on the targets, with the OBC condition producing more target visits than the other occlusion conditions. It should be noted that the OBC condition also contained more occlusions than the two other occlusion conditions. Moreover, participants blinked less when tracking targets involved in occlusions than nonoccluded targets. The effect reflects a higher need for visual sampling in the occlusion conditions – an interpretation compatible with the fixation data reviewed above. The reduction in blink rate was less noticeable in the OC condition than the other two conditions, perhaps reflecting the fact that a possibility for target-distractor swapping did not exist in this condition. Finally, pupils dilated from baseline more when tracking targets involved in occlusion than when tracking nonoccluded targets. The OBC condition was associated with greater pupil dilation than the two other occlusion conditions. As pupil size is shown to reflect cognitive effort, these results suggest that tracking is cognitively more demanding when targets are occasionally occluded, especially when targets are involved in frequent occlusions containing both OB and OC occlusions. Despite the increased cognitive efforts in visual sampling during tracking of occasionally occluded objects, tracking accuracy was poorer for the occlusion conditions than for the NO condition, with the OBC condition associated with the poorest tracking performance.

The detrimental effect of occlusion observed in tracking accuracy is generally in line with what Zelinsky and Todor (2010) report, although the exact nature of the effect is somewhat different across the two studies. Zelinsky and Todor observed it only with four targets, but we found it even with two targets, although the effect was more robust with four targets in the OC and OBC conditions. It should be noted that Zelinsky and Todor investigated effects of OB occlusion, that is, a condition analogous to our OB condition. Viswanathan and Mingolla (1998) also considered effects of OB occlusion. However, their question was different from ours. They mainly studied the effect of depth cues while tracking simple circles moving with uniform speed through OB occlusions. They added shades to objects to induce depth cues and the perception that one object is in front of another one. Performance in this condition is reported to be better than in the condition with no depth cues. They did not manipulate set size (all trials contained 5 targets) or compare the results with the NO condition. Finally, our OC condition is similar to the “occlusion” condition in the Scholl and Pylyshyn (1999) study. In their study, targets (solid squares) moved behind rectangular occluders with visible borders without any OB occlusions (all trials contained 4 targets). Tracking accuracy for this condition was not significantly worse than for the NO condition, although there was a trend in that direction (only 15 participants were included in their study sample). They conclude that the MTT mechanism allows for occlusions and supports tracking of targets despite their temporary disappearance – a conclusion at odds with the studies reviewed above.

It is worth noting that the target set size effects observed in the present study resemble more closely the results obtained for multiple identity tracking with distinct identities than for tracking identical objects (Oksama & Hyönä, 2016). In the present study, participants blinked less and made more but shorter fixations when tracking four than two targets. Moreover, they made more target visits when tracking four than two targets. Finally, pupils dilated more from baseline when tracking four rather than two targets. Oksama and Hyönä (2016) obtained analogous set size effects for multiple identity tracking, but the set size effect in fixation frequency, fixation duration, and target visits were absent when tracking multiple identical objects (e.g. black circles). Thus, this suggests that although our stimulus objects (larvae) look alike, their identities are distinguishable when in motion (e.g. by their unique ways of moving about in the container) and were tracked as unique identities.

Finally, it should be noted that the present results were obtained with naturally occurring larva motion. The stimuli were two-dimensional with a simple background and a fixed number of objects, which resembles the synthetic stimuli commonly used in the literature. Therefore, our work can be considered a step toward generalization of previous results on artificial motion scenarios. Yet, it is not clear to what extent the results generalize to other types of motion. The studied larva motion is characterized by taking place in bursts interspersed with occasional pauses. In other words, the speed is not constant within and between targets, as has been the case in many previous MOT studies. Future studies will show to what extent the observed effects of occlusion on eye behavior are specific to larva motion.

## Conclusions

When tracking multiple moving objects, observers make anticipatory saccades toward the to-be-occluded targets in real-life scenarios. The present study demonstrated that these saccades facilitate tracking in cases when the target is occluded by another moving object, presumably by helping to avoid confusing the target with another moving object. The timing of RSs is critical, as they improved target tracking only when they were executed within the 500 ms time window preceding the occlusion onset. On the other hand, they are ineffective when directed to targets that are about to be occluded by a stationary occluder, which make the target completely or mostly invisible. In general, occlusions in MTT increase cognitive effort, as indexed by enlarged pupil size. In addition, when tracking occluded targets, subjects make more fixations with shorter duration and less blinks to be able to keep track of targets in such challenging situation. Yet, the presence of occlusions deteriorates tracking accuracy.

## Supplementary Material

Supplement 1

Supplement 2

Supplement 3

Supplement 4

## Footnotes

1
https://github.com/Xiao-ying/-moving-zebrafish-larvae-segmentation-dataset-/tree/master/Data.

2 The results are very similar when the proportion of correctly tracked targets was used as the measure of accuracy.

3
https://github.com/wkentaro/labelme.
